# Prognostic Significance of Severe Vitamin D Deficiency in Patients with Primary Sclerosing Cholangitis

**DOI:** 10.3390/nu15030576

**Published:** 2023-01-22

**Authors:** Maryam Ebadi, Elora Rider, Catherine Tsai, Sarah Wang, Ellina Lytvyak, Andrew Mason, Aldo J. Montano-Loza

**Affiliations:** Liver Unit, Division of Gastroenterology, University of Alberta Hospital, Edmonton, AB T6G 2X8, Canada

**Keywords:** chronic cholestasis, low levels of serum vitamin D, liver-related mortality, progression of cirrhosis

## Abstract

Vitamin D deficiency has been linked with adverse events in various liver diseases. The present study aimed to recognize the association between severe vitamin D deficiency and disease progression, hepatobiliary malignancies, liver-related mortality, and the need for liver transplantation in primary sclerosing cholangitis (PSC). Patients with a diagnosis of PSC (*n* = 354), followed by the autoimmune liver disease clinic at the University of Alberta, were included. Patients with vitamin D levels < 25 nmol/L were defined as severely deficient. Univariate and multivariate analyses were conducted using the Cox proportional hazards regression models. The mean vitamin D level was 59 ± 2 nmol/L, and 63 patients (18%) had a severe vitamin D deficiency. Patients with a severe vitamin D deficiency were 2.5 times more likely to experience hepatobiliary malignancies (HR 2.55, 95% CI, 1.02–6.40, *p* = 0.046). A severe vitamin D deficiency at diagnosis (HR 1.82, 95% CI, 1.05–3.15, *p* = 0.03) and persistent deficiencies over time (HR 2.26, 95% CI, 1.17–4.37, *p* = 0.02) were independently associated with a higher risk of poor clinical liver outcomes. A severe vitamin D deficiency at diagnosis and persistent deficiency at longitudinal assessments were associated with liver-related mortality or the need for liver transplantation.

## 1. Introduction

Primary sclerosing cholangitis (PSC) is a chronic cholestatic liver disease characterized by progressive biliary inflammation and fibrosis of the intra- and extra-hepatic ducts [1]. In general, the prognosis is poor in the long term for this patient population due to the lack of medical treatments and the risk of hepatobiliary malignancies, episodes of cholangitis, and decompensated cirrhosis. Moreover, a significant percentage of patients have concurrent inflammatory bowel disease (IBD), either ulcerative colitis, or Crohn’s disease [2]. Commonly, PSC patients have a mean time to death or liver transplantation of 10 to 22 years after diagnosis [3].

Vitamin D is a secosteroid involved in anti-inflammatory, anti-proliferative, and anti-fibrotic pathways. Given the major role of the liver in converting cholecalciferol to the main circulating form, 25-hydroxyvitamin D, chronic liver diseases might consequently affect the production of the vitamin D active form [4]. Moreover, vitamin D plays a critical function in the natural history and development of liver diseases (reviewed in [5]). Therefore, vitamin D deficiency in patients with chronic liver diseases may be related to the liver damage and impaired vitamin D3 hydroxylation.

An association between vitamin D deficiency and the pathogenesis of autoimmune liver diseases has been suggested in recent studies [6]. We previously described, in a cohort of patients with autoimmune hepatitis, that severe vitamin D deficiency (<25 nmol/L) is associated with the progression of the disease and a higher risk of liver-related events (liver transplantation or mortality) [7]. In patients with primary biliary cholangitis (PBC), links between vitamin D deficiency and the progression of the disease [8], incomplete response to ursodeoxycholic acid (UDCA) [9,10], cirrhosis progression, an increased need for liver transplantation, and a higher risk of mortality were described [10]. In a previous study including 54 patients with PSC, vitamin D serum level was inversely associated with the stage of fibrosis at the time of diagnosis [11]. The association between PSC severity and vitamin D levels of serum was stated in earlier studies. Serum levels of vitamin D were lower in patients with late histological stages of PSC (stages 3 and 4) when compared to patients in stages 1 and 2 [12].

The etiology of PSC is not entirely elucidated; however, regulatory T-cell impairment may be important in its pathogenesis and progression [13]. Vitamin D modulates circulating T regulatory cell numbers and phenotypes [14]. Given the importance of vitamin D in the modulation of inflammatory and/or immune-mediated pathways, vitamin D status may impact the PSC course; however, this link has been evaluated in a limited number of studies.

The objectives of this study were to identify the prevalence of severe vitamin D deficiency in patients with PSC and to investigate its association with cirrhosis progression, hepatobiliary malignancies, liver-related mortality, and the need for liver transplantation.

## 2. Materials and Methods

### 2.1. Patient Population

This retrospective study was reviewed and accepted by the Institutional Review Board of the University of Alberta. Patients diagnosed with PSC, according to the European Association for the Study (EASL) guidelines [3] and the American Association for Study of the Liver (AASLD) practice guidance [15], were included. Patients assessed and monitored by the Autoimmune Liver Disease Clinic at the University of Alberta, Edmonton, Canada from 1972–2019 were initially included (*n* = 455). Patients were excluded from the study if they did not have vitamin D measurements available at the time of diagnosis (*n* = 101), leaving 354 included patients.

### 2.2. Clinical and Laboratory Evaluations

Liver function tests, including serum alanine aminotransferase (ALT), alkaline phosphatase (ALP), bilirubin, and international normalized ratio (INR) were evaluated at each clinical visit. For the purpose of this study, biochemical parameters measured at the time of vitamin D assessment were used for analysis.

Vitamin D levels were evaluated at diagnosis, or the first clinic visit in the Core Laboratory, University of Alberta Hospital. This procedure assesses the 25-hydroxyvitamin D of the serum. In line with chronic liver diseases [16,17,18,19,20] and other non-liver chronic diseases [21], severe vitamin D deficiency was described as vitamin D < 25 nmol/L. Clinical and biochemical data at the time of vitamin D assessment were used to calculate the Mayo Score [22].

### 2.3. Cirrhosis Evaluation at Diagnosis or Follow-Up

Cirrhosis at diagnosis or development of cirrhosis in patients without this finding at diagnosis was identified based on the presence of histological findings in liver biopsies, elevated FibroScan score (>14 kPa), radiological characteristics of the cirrhotic liver by ultrasound (US), computerized tomography (CT) or magnetic resonance imaging (MRI), or portal hypertension complications (ascites, variceal bleeding, hepatic encephalopathy). Patients had MRIs to look for high-grade strictures and liver US every year to look for gallbladder polyps, as per guideline recommendations for surveillance of hepatobiliary cancers [23]. Radiological diagnosis of cirrhosis was established with liver dysmorphia plus signs of portal hypertension, such as splenomegaly, ascites, or umbilical vein recanalization.

### 2.4. High-Grade Strictures and Hepatobiliary Malignancy

High-grade strictures were defined as a biliary stricture on MRI/MRCP with a >75% reduction of duct diameter in the common bile duct or hepatic ducts, and these patients underwent a diagnostic work-up with endoscopic retrograde cholangiopancreatography (ERCP) with or without cholangioscopy as an investigation for cholangiocarcinoma. Patients with high-grade strictures with signs or symptoms of obstructive cholestasis and/or bacterial cholangitis (Relevant stricture) underwent ERCP for the consideration of endoscopic therapy with balloon dilation. High-grade strictures were recorded at diagnosis and during follow-ups. The types of hepatobiliary malignancies included in this study were cholangiocarcinoma, hepatocellular carcinoma, and gallbladder cancer. This is in line with the current guidelines for surveillance recommendations of hepatobiliary cancers in patients with PSC [3,15].

### 2.5. Liver-Related Outcomes

Liver-related outcomes were defined as liver-related mortality or liver transplantation. Mortality related to liver dysfunction, hepatobiliary malignancies, sepsis related to cholangitis or portal hypertension, complications such as variceal bleeding, hepatic encephalopathy, or hepatorenal syndrome were stated to be a liver-related mortality.

### 2.6. Statistical Analysis

Continuous variables were reported as means with standards error of the mean (S.E.M), and the differences in the means were compared using the unpaired *t*-test. Relationships between categorical variables were confirmed using Fisher’s exact test. Vitamin D predictive association was assessed as a continuous variable followed by comparing patients with and without a severe deficiency in 25-OH vitamin D (< and ≥25 nmol/L).

An association between vitamin D and outcomes was investigated using the univariate and multivariate Cox proportional hazards regression models, with the results presenting as hazard ratios (HR) with 95% confidence intervals (CI). Factors with a *p*-value <0.10 in the univariate analysis were incorporated into the final multivariate model. Overall survival estimates and survival probabilities were obtained by applying the Kaplan–Meier curves, and the comparison between survival curves was conducted using the Log Rank (Mantel–Cox) test. Patients who were missed throughout follow-ups and patients who were constantly monitored in the clinic were censored at the time of study termination. Statistical analyses were conducted using SPSS (SPSS for Windows, version 26.0, SPSS, Chicago, IL, USA), and a *p*-value less than 0.05 was considered to be a statistically significant difference.

## 3. Results

### 3.1. Patient Characteristics

Of the 354 patients with PSC evaluated in this study, 118 (33%) were female. The mean age at diagnosis was 35 ± 1 years, and the mean body mass index (BMI) was 25 ± 0.3 kg/m^2^. Cirrhosis at the time of diagnosis was identified in 38 patients (11%). Inflammatory bowel disease was present in 75% of patients, and high-grade strictures occurred in 40% of the patients.

The mean vitamin D level was 59 ± 2 nmol/L (range, 4–165 nmol/L), and 63 patients (18%) had a severe vitamin D deficiency (<25 nmol/L). Patients with a severe vitamin D deficiency had lower vitamin D levels regardless of the season. Table 1 presents the clinical and biochemical characteristics of patients with and without a severe vitamin D deficiency (< 25 vs. ≥ 25 nmol/L). Serum levels of ALT (166 ± 29 vs. 83 ± 5, *p* < 0.001), bilirubin (116 ± 15 vs. 36 ± 3, *p* < 0.001), IgG (1.3 ± 0.4 vs. 0.6 ± 0.1, *p* = 0.008), INR (1.3 ± 0.04 vs. 1.2 ± 0.02, *p* = 0.005), and Mayo Score (1.40 ± 0.2 vs. 0.53 ± 0.1, *p* < 0.001) were significantly higher in patients with PSC and a severe vitamin D deficiency when compared to their counterparts. Other clinical parameters including age at diagnosis, sex, IBD, BMI, development of high-grade strictures, history of ascites and variceal bleeding, and the frequency of cirrhosis at baseline did not significantly differ between groups with different vitamin D statuses.

### 3.2. Hepatobiliary Malignancy

**Figure 1:**
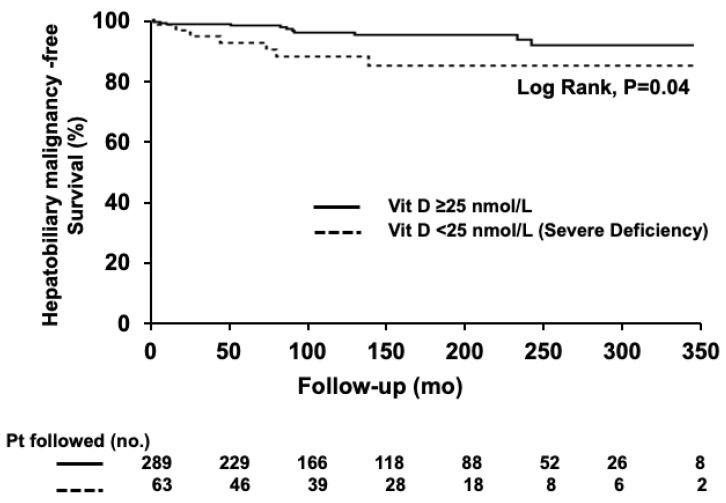
Hepatobiliary malignancy-free survival according to the status of severe vitamin D deficiency.

The hepatobiliary malignancy rate was 6% in this study, with cholangiocarcinoma being the most common. There was a statistical trend regarding hepatobiliary malignancies occurring more commonly in patients with a severe vitamin D deficiency than in patients without a severe deficiency (11% vs. 5%, *p* = 0.07; Table 1). Patients with a severe vitamin D deficiency were 2.5 times more likely to experience hepatobiliary malignancies over the duration of the follow-up (HR 2.55, 95% CI, 1.02–6.40, *p* = 0.046). However, this association was no longer significant when adjusting for the bilirubin level (HR 1.86, 95% CI, 0.65–5.31, *p* = 0.25), which was another independent predictor of hepatobiliary malignancies. The 5- and 10-year probabilities of hepatobiliary malignancy-free survival were 93% and 89% in patients with severe vitamin D deficiency, compared to 98% and 96% in patients without severe deficiency (Log-rank, *p* = 0.04, Figure 1).

Kaplan–Meier curves with the log-rank test were applied for the estimation of survival rate over time and comparison between curves, respectively. Hepatobiliary malignancy-free survival was shorter in patients with a severe vitamin D deficiency (Log-rank, *p* = 0.04).

### 3.3. Development of Cirrhosis

Of 316 patients without cirrhosis at diagnosis, 151 (44%) developed cirrhosis during the follow-up. The median time to develop cirrhosis was 179 months (95% CI, 154–204). Serum vitamin D level as a continuous variable was only associated with cirrhosis development in univariate analysis (HR 0.99, 95% CI, 0.99–0.999, *p* = 0.02; Table 2). There was no association between severe vitamin D deficiency at presentation and the risk of developing cirrhosis.

### 3.4. Liver-Related Mortality or Liver Transplantation

Over a median follow-up period of 153 months (95% CI, 130–177), 24 deaths were attributed to liver-related disease, and 137 patients underwent a liver transplantation. In patients who received a liver transplant, the mean level of serum vitamin D was lower (54 ± 3 vs. 63 ± 2 nmol/L, *p* = 0.01). A significantly higher frequency of poor clinical liver outcomes (liver transplantation or liver-related death) was observed in patients with a severe vitamin D deficiency than in patients without a severe deficiency (62% vs. 42%, *p* = 0.005; Table 1).

**Figure 2:**
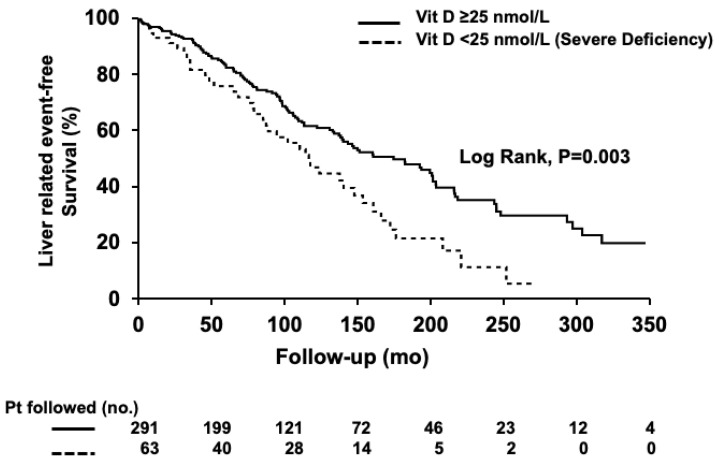
Liver-related event-free survival according to severe vitamin D deficiency status.

Serum levels of ALT, ALP, bilirubin, and creatinine; sex; ascites; variceal bleeding; BMI ≥ 25 kg/m^2^; development of high-grade strictures; hepatobiliary malignancy; and both the serum vitamin D level and severe vitamin D deficiency (Table 3) were associated with poor clinical liver outcomes in univariate analysis. Serum vitamin D level as a continuous variable was associated with poor clinical liver outcomes (HR 0.99, 95% CI, 0.99–0.999, *p* = 0.01) in univariate, but not in the multivariate, analysis (HR 0.995, 95% CI, 0.99–1.002, *p* = 0.18). Severe vitamin D deficiency, however, was independently associated with a higher risk of poor clinical liver outcomes (HR 1.82, 95% CI, 1.05–3.15, *p* = 0.03, Table 3) after adjusting for other confounding factors in multivariate analysis. In patients with severe vitamin D deficiency, liver-related events-free survival was shorter when compared to patients without severe deficiency (119 months; 95% CI, 81–156 vs. 185 months; 95% CI, 146–224, *p* = 0.003, log-rank test; Figure 2). The Mayo risk score was not included in the multivariate analysis to avoid the overestimation of the model due to the collinearity of the variables.

Survival was predicted using Kaplan–Meier curves, and the comparison between curves was made using the log-rank test. Liver-related event-free survival was shorter in patients with a severe vitamin D deficiency (Log-rank, *p* = 0.003).

**Figure 3:**
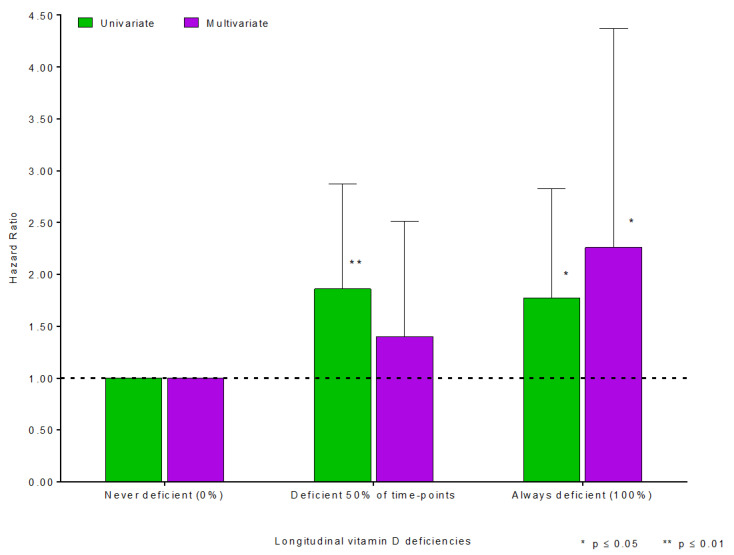
Hazard ratios of the association between longitudinal severe vitamin D deficiency and liver-related events (mortality and transplantation).

Following the vitamin D assessment at baseline and prior to supplementation initiation, at least three further vitamin D levels were also available in most of the patients. The mean time between the baseline assessment and the second vitamin D measurement was 20 months. The third and fourth measurements were available at a mean time of 18 and 13 months from the second and third assessments, respectively. Using these values, patients were divided into three groups regarding longitudinal severe vitamin D deficiencies: always deficient, less than 50% of the time-points deficient, and never deficient. Patients with persistent severe vitamin D deficiencies at all time points (10% of the population) had more than two-fold higher risk of poor clinical liver outcomes after adjusting for other confounding predictors (HR 2.26, 95% CI, 1.17–4.37, *p* = 0.02; Table 4, Figure 3).

The reference group is never deficient patients, and the vertical lines denote 95% confidence intervals around the hazard ratio estimates. Hazard ratios are adjusted for the serum levels of ALT, ALP, bilirubin, and creatinine; sex; ascites; variceal bleeding; BMI ≥25 kg/m^2^; development of high-grade strictures; and hepatobiliary malignancy.

### 3.5. Response to Vitamin D Supplementation

Regardless of vitamin D status at diagnosis, 195 patients were prescribed various vitamin D regimens with doses ranging from megadoses of 500,000 international units (IUs) to 50,000 IU of vitamin D2 or 4000 to 400 IUs of D3. The mean level of vitamin D was 59 ± 2 nmol/L at baseline, which improved to 77 ± 3 nmol/L in supplemented patients (*p* < 0.00; Table 5). Of the 63 severely deficient patients, 34 patients (54%) were supplemented with vitamin D. Among those, five patients continued to have a severe deficiency following supplementation, and poor clinical liver outcomes were reported in three of them (60%).

## 4. Discussion

Severe vitamin D deficiency presented in 18% of patients with PSC at diagnosis and was correlated with a higher frequency of liver-related events and hepatobiliary malignancies. We also noticed that persistent severe vitamin D deficiency was common among 10% of the patients. This persistent deficiency was associated with a two-times-higher risk of adverse events. Overall, there is limited data on the vitamin D status of patients with PSC.

The underlying mechanism of vitamin D deficiency in liver disease has not been clearly recognized, though it is assumed to be multifactorial and related to diminished intestinal absorption, nutritional deficiency, inadequate sun exposure, and limited hydroxylation by hepatocytes [24]. No association was found between season of vitamin D assessment and its level in this high-latitude part of Canada. This is in line with the results of former studies in patients with cirrhosis [17] and hepatocellular carcinoma [20], demonstrating that it is not variations in sun exposure, but rather altered hepatic hydroxylation by the liver that is the main factor associated with vitamin D deficiency.

Although the clinical implications of vitamin D deficiency in the prognosis of several liver diseases have been suggested (reviewed in [25,26]), it has not been recognized whether a vitamin D deficiency is a cause, trigger, or outcome. A vitamin D deficiency could be the result of the severe cholestasis, and the progression of the PSC would induce the severe vitamin D deficiency. Moreover, the possibility of a vitamin D deficiency as a pre-existing undetermined feature contributing to PSC development prior to its diagnosis cannot be neglected. Given the immune-mediated mechanisms in the initiation and progression of PSC and the immunomodulatory and anti-inflammatory properties [25] of vitamin D, the contribution of a vitamin D deficiency to the progression of liver damage might be postulated. Additionally, the association between a severe vitamin D deficiency and poor outcomes in this study may suggest its role as a marker of advanced liver disease.

Besides vitamin D, the serological levels of free light chains (FLCs) are considered potential biomarkers in immune-mediated diseases. Low levels of serum vitamin D have been reported in patients with chronic hepatitis C virus (HCV) infections. However, the role of vitamin D as a prognostic biomarker has not been confirmed in HCV patients, and, rather, FLCs might play a more important role in B cell activation in the course of HCV infection [27]. The importance of FLCs in PSC prognosis needs to be investigated in future studies.

A well-known independent prognostic parameter for PSC, bilirubin, was not significant in the multivariate analysis for liver-related outcomes. It might be related to the fact that intrinsic liver function (bilirubin, albumin, platelets) is important in predicting an immediate outcome [28]. However, liver-related event-free survival in this study was defined as the duration from the date of diagnosis to the date of liver-related events. There was a trend for serum ALP in multivariate analysis to predict liver-related events. Although the prognostic importance of serum ALP as a long-term predictor of clinical events in PSC was established [28], the poor prognosis of malignancies in predicting longer term outcomes should not be neglected [29].

In this study, the frequency of hepatobiliary malignancy was higher in patients with PSC and severe vitamin D deficiency. The association between vitamin D status and various types of malignancies has been the focus of prior studies. This might be related to vitamin D properties, including anti-proliferative, anti-angiogenic, proapoptotic, and prodifferentiative effects [30]. However, the main impact of vitamin D in hepatocellular carcinoma seems to be the inhibition of cell proliferation [31]. In a German prospective cohort study, a severe vitamin D deficiency was associated with advanced stages of hepatocellular carcinoma and a higher mortality risk. This association was independent of the MELD score and alpha-fetoprotein levels [20]. Although the results of observational studies regarding the association between vitamin D status and liver cancer development are promising, they are still inadequate [31]. In a retrospective study of cholangiocarcinoma patients, vitamin D intake was associated with shorter disease-free survival in patients who underwent surgery [32]. This demonstrates the necessity for future prospective studies with a larger number of patients to investigate the association between vitamin D status and intake with hepatobiliary malignancy in patients with PSC.

IBD was noted in more than 70% of PSC patients in this study. With the considerable overlap between PSC and IBD, comparisons related to the impacts of vitamin D status on several outcomes might be probable. In patients with Crohn’s disease, the frequency of vitamin D deficiency was estimated to be as high as 70% [30]. PSC occurrence was an independent predictor of vitamin D deficiency (≤50 nmol/L) in patients with Crohn’s disease and ileal pouch–anal anastomosis (odds ratio: 7.56; 95% CI: 2.39–24.08; *p* = 0.001) [31].

Interestingly, we found that the presence of IBD is associated with a protective effect for cirrhosis development. Similar to our study, previous experiences suggested concomitant IBD may improve the outcome of PSC [33]. However, the data regarding this association are still conflicting, as other studies suggest concomitant IBD may be related to worse outcomes in PSC, with a higher risk of liver transplant requirement, mortality, and hepatobiliary cancer risk [34,35].

Given the poor prognosis of PSC and the lack of effective treatment options, the potential therapeutic application of vitamin D should be investigated in prospective clinical trials. Due to a lack of guidelines on vitamin D supplementation in PSC, this retrospective cohort presented data on variable doses and durations of supplementation, which limits our ability to make a conclusive statement. Moreover, in cholestatic diseases, intestinal malabsorption of vitamin D due to scarce concentrations of intraluminal bile acid might contribute to vitamin D deficiency [36], and, therefore, sporadic intramuscular injections of vitamin D rather than oral administration were suggested as an effective strategy [37].

We acknowledge some weaknesses of the current research, as it was a single-center study with a limited number of patients. Due to the retrospective nature, some patients might not have had proper screening, especially those recruited in the 1970s or 1980s. Moreover, we cannot conclude whether a severe vitamin D deficiency itself results in adverse events or if it mainly suggests more advanced stages of liver disease. Although dietary intake of vitamin D was not evaluated in this study, in geographical locations and seasons with low sun exposure, vitamin D supplementation is inevitable due to restricted dietary sources. We acknowledge that fluctuations in disease progression over the time period of a study is a potential confounding factor. Biochemical variables in this study were collected at the time of vitamin D assessment, and fluctuations in their levels, especially in the early stages of diagnosis, may limit their prognostic values. Finally, genetic variability in the metabolism of vitamin D needs to be considered [38].

## 5. Conclusions

In conclusion, 18% of patients with PSC had severe vitamin D deficiency at diagnosis which was associated with hepatobiliary malignancy. A low level of serum vitamin D at diagnosis also appeared to be an independent predictor of cirrhosis development; however, this was only in univariate analysis. A severe vitamin D deficiency at presentation and persistent deficiency at longitudinal assessments were significantly associated with liver-related mortality or the need for liver transplantation. Patients with PSC should undergo a routine assessment of vitamin D levels, which might be a prognostic biomarker. Lastly, prospective clinical trials should explore the influence of vitamin D supplementation on the progression of the disease and outcomes.

## Figures and Tables

**Table 1:** Clinical and Biochemical Characteristics Related to Vitamin D Deficiency in PSC.

Characteristics	All (*n* = 354)	<25 nmol/L (*n* = 63)	≥25 nmol/L (*n* = 291)	*p*-Value
Features collected at the time of vitamin D assessment				
Diagnosis age, years	35 ± 1	36 ± 2	35 ± 1	0.73
Sex, male	236 (67)	48 (76)	188 (65)	0.08
ALT, U/L	97 ± 7	166 ± 29	83 ± 5	<0.001
ALP, U/L	315 ± 15	376 ± 33	307 ± 15	0.06
Cirrhosis at diagnosis	38 (11)	7 (11)	31 (11)	1.00
Bilirubin, µmol/L	48 ± 8	116 ± 15	36 ± 3	<0.001
Serum IgG, g/L	0.7 ± 0.1	1.3 ± 0.4	0.6 ± 0.1	0.008
Creatinine, µmol/L	85 ± 14	87 ± 6	79 ± 4	0.38
INR	1.1 ± 0.03	1.3 ± 0.04	1.2 ± 0.02	0.005
Platelet (10^9^/L)	238 ± 14	231 ± 18	236 ± 7	0.77
IBD	271 (77)	47 (75)	224 (77)	0.74
BMI, kg/m^2^	25 ± 0.3	26 ± 0.7	25 ± 0.3	0.58
BMI ≥ 25 kg/m^2^	137 (39)	22 (44)	115 (46)	0.88
Mayo Score	0.70 ± 0.1	1.40 ± 0.2	0.53 ± 0.1	<0.001
Serum vitamin D level, nmol/L	59 ± 2	16 ± 0.7	69 ± 2	<0.001
Serum vitamin D level, nmol/L				
Spring–Summer	64 ± 3	16 ± 1	73 ± 3	<0.001
Fall–Winter	54 ± 2	16 ± 1	64 ± 2	<0.001
Seasonal variation	179 (51)	26 (41)	153 (53)	
Spring–Summer				0.13
Fall–Winter	175 (49)	37 (59)	138 (47)	
Events occurring during the follow-up				
Ascites	65 (18)	17 (27)	48 (17)	0.07
Variceal bleeding	15 (4)	2 (3)	13 (5)	1.00
Liver-related events	161 (45)	39 (62)	122 (42)	0.005
High-grade strictures	143 (40)	24 (39)	119 (43)	0.67
Hepatobiliary malignancy	21 (6)	7 (11)	14 (5)	0.07

Abbreviations: ALT, alanine aminotransferase; ALP, alkaline phosphatase; BMI, body mass index; IBD, inflammatory bowel disease; INR, international normalized ratio; PSC, primary sclerosing cholangitis. Numbers in parentheses show percentages. Continuous variables are presented as mean ± S.E.M.

**Table 2:** Features Associated with Cirrhosis Development in PSC.

	Univariate		Multivariate	
Characteristics	HR (95% CI)	*p*-Value	HR (95% CI)	*p*-Value
Diagnosis age, years	1.001 (0.99–1.01)	0.83		
Sex, male	1.09 (0.76–1.56)	0.63		
ALT, U/L	1.002 (1.00–1.003)	0.01	1.001 (0.999–1.002)	0.57
ALP, U/L	1.001 (1.001–1.002)	<0.001	1.001 (1.00–1.002)	<0.001
Bilirubin, µmol/L	1.001 (0.999–1.003)	0.25		
Serum IgG, g/L	0.998 (0.60–1.67)	0.99		
INR	0.94 (0.63–1.40)	0.75		
Platelet (10^9^/L)	0.999 (0.997–1.00)	0.07	0.998 (0.997–1.00)	0.04
IBD	0.67 (0.45–0.99)	0.04	0.56 (0.36–0.87)	0.01
BMI ≥ 25 kg/m^2^	0.86 (0.60–1.23)	0.39		
High-grade strictures	0.99 (0.99–1.002)	0.14		
Serum vitamin D level, nmol/L	0.99 (0.99–0.999)	0.02	0.995 (0.99–1.00)	0.11
Severe vitamin D deficiency (< 25 nmol/L)	1.35 (0.90–2.03)	0.14		

Abbreviations: ALT, alanine aminotransferase; ALP, alkaline phosphatase; BMI, body mass index; IBD, inflammatory bowel disease; INR, international normalized ratio; PSC, primary sclerosing cholangitis. Cox proportional hazard model was applied to estimate HRs and *p*-values.

**Table 3:** Parameters Associated with Liver-Related Events (Mortality and Transplantation) in PSC.

	Univariate		Multivariate	
Characteristics	HR (95% CI)	*p*-Value	HR (95% CI)	*p*-Value
Diagnosis age, years	1.003 (0.99–1.02)	0.58		
Sex, male	1.76 (1.23–2.53)	0.002	1.75 (1.03–2.99)	0.04
ALT, U/L	1.002 (1.00–1.003)	0.005	1.00 (0.999–1.003)	0.21
ALP, U/L	1.001 (1.00–1.001)	0.01	1.001 (1.00–1.001)	0.08
Cirrhosis at diagnosis	1.09 (0.71–1.67)	0.69		
Bilirubin, µmol/L	1.003 (1.001–1.004)	<0.001	1.001 (0.999–1.003)	0.45
Serum IgG, g/L	1.39 (0.90–2.13)	0.14		
Creatinine, µmol/L	1.002 (1.00–1.003)	0.05	1.002 (1.00–1.003)	0.12
INR	1.05 (0.74–1.49)	0.77		
Platelet (10^9^/L)	0.999 (0.998–1.00)	0.17		
Ascites	1.70 (1.21–2.38)	0.002	1.40 (0.86–2.28)	0.17
Variceal bleeding	2.37 (1.37–4.10)	0.002	1.36 (0.62–3.01)	0.44
IBD	1.30 (0.86–1.96)	0.22		
BMI ≥ 25 kg/m^2^	0.74 (0.52–1.04)	0.08	0.75 (0.47–1.18)	0.21
High-grade strictures	1.58 (1.14–2.20)	0.007	1.30 (0.85–2.01)	0.23
Hepatobiliary malignancy	2.03 (1.22–3.38)	0.006	2.62 (1.35–5.09)	0.004
Serum vitamin D level, nmol/L	0.99 (0.99–0.999)	0.01		
Severe vitamin D deficiency (< 25 nmol/L)	1.74 (1.21–2.51)	0.003	1.82 (1.05–3.15)	0.03

Abbreviations: ALT, alanine aminotransferase; ALP, alkaline phosphatase; BMI, body mass index; IBD, inflammatory bowel disease; INR, international normalized ratio; PSC, primary sclerosing cholangitis. Cox proportional hazard model was applied to estimate HRs and *p*-values.

**Table 4:** Association between Longitudinal Severe Vitamin D deficiency and Liver-Related Events (Mortality and Transplantation).

	Univariate		Multivariate	
Characteristics	HR (95% CI)	*p*-Value	HR (95% CI)	*p*-Value
Sex, male	1.76 (1.23–2.53)	0.002	1.87 (1.09–3.21)	0.02
ALT, U/L	1.002 (1.00–1.003)	0.005	1.001 (1.00–1.003)	0.11
ALP, U/L	1.001 (1.00–1.001)	0.01	1.001 (1.00–1.001)	0.17
Bilirubin, µmol/L	1.003 (1.001–1.004)	<0.001	1.001 (0.999–1.003)	0.27
Creatinine, µmol/L	1.002 (1.00–1.003)	0.05	1.001 (0.999–1.003)	0.20
Ascites	1.70 (1.21–2.38)	0.002	1.34 (0.82–2.17)	0.24
Variceal bleeding	2.37 (1.37–4.10)	0.002	1.29 (0.58–2.86)	0.54
BMI ≥ 25 kg/m^2^	0.74 (0.52–1.04)	0.08	0.76 (0.49–1.20)	0.25
High-grade strictures	1.58 (1.14–2.20)	0.007	1.21 (0.78–1.86)	0.40
Hepatobiliary malignancy	2.03 (1.22–3.38)	0.006	2.73 (1.41–5.26)	0.003
Longitudinal vitamin D deficiencies				
Never	Ref		Ref	-
50% of time-points	1.83 (1.16–2.87)	0.009	1.40 (0.78–2.51)	0.26
Always deficient	1.77 (1.11–2.83)	0.02	2.26 (1.17–4.37)	0.02

Abbreviations: ALT, alanine aminotransferase; ALP, alkaline phosphatase; BMI, body mass index. HRs and *p*-values were calculated by Cox proportional hazard analysis.

**Table 5:** Vitamin D characteristics according to the deficiency status.

Characteristics of Vitamin D	All (*n* = 354)	<25 nmol/L (*n* = 63)	≥25 nmol/L (*n* = 291)	*p*-Value
Baseline levels	59 ± 2	16 ± 0.7	69 ± 2	<0.001
Follow-up levels	73 ± 2	44 ± 5	78 ± 2	<0.001
Supplementation	195 (56)	34 (54)	161 (56)	0.78
Levels after supplementation	77 ± 3	57 ± 9	82 ± 3	0.002

## Data Availability

The datasets generated and analyzed during the current study are not publicly available but are available from the corresponding author on reasonable request.

## References

[1] Chapman R., Fevery J., Kalloo A., Nagorney D.M., Boberg K.M., Shneider B., Gores G.J. (2010). American Association for the Study of Liver D: Diagnosis and management of primary sclerosing cholangitis. Hepatology.

[2] Karlsen T.H., Folseraas T., Thorburn D., Vesterhus M. (2017). Primary sclerosing cholangitis-a comprehensive review. J. Hepatol..

[3] European Association for the Study of the Liver (2022). Electronic address eee, European Association for the Study of the L: EASL Clinical Practice Guidelines on sclerosing cholangitis. J. Hepatol..

[4] Kitson M.T., Roberts S.K. (2012). D-livering the message: The importance of vitamin D status in chronic liver disease. J. Hepatol..

[5] Konstantakis C., Tselekouni P., Kalafateli M., Triantos C. (2016). Vitamin D deficiency in patients with liver cirrhosis. Ann. Gastroenterol..

[6] Czaja A.J., Montano-Loza A.J. (2019). Evolving Role of Vitamin D in Immune-Mediated Disease and Its Implications in Autoimmune Hepatitis. Dig. Dis. Sci..

[7] Ebadi M., Bhanji R.A., Mazurak V.C., Lytvyak E., Mason A., Czaja A.J., Montano-Loza A.J. (2019). Severe vitamin D deficiency is a prognostic biomarker in autoimmune hepatitis. Aliment. Pharmacol. Ther..

[8] Agmon-Levin N., Kopilov R., Selmi C., Nussinovitch U., Sanchez-Castanon M., Lopez-Hoyos M., Amital H., Kivity S., Gershwin E.M., Shoenfeld Y. (2015). Vitamin D in primary biliary cirrhosis, a plausible marker of advanced disease. Immunol. Res..

[9] Guo G.Y., Shi Y.Q., Wang L., Ren X., Han Z.Y., Guo C.C., Cui L.N., Wang J.B., Zhu J., Wang N. (2015). Serum vitamin D level is associated with disease severity and response to ursodeoxycholic acid in primary biliary cirrhosis. Aliment. Pharmacol. Ther..

[10] Ebadi M., Ip S., Lytvyak E., Asghari S., Rider E., Mason A., Montano-Loza A.J. (2022). Vitamin D Is Associated with Clinical Outcomes in Patients with Primary Biliary Cholangitis. Nutrients.

[11] Galante A., Wiegard C., Weiler-Normann C., Quaas A., Lohse A.W., Schramm C. (2014). Vitamin D deficiency in primary sclerosing cholangitis is related to histological fibrosis stage at the time of diagnosis. Z. Gastroenterol..

[12] Jorgensen R.A., Lindor K.D., Sartin J.S., LaRusso N.F., Wiesner R.H. (1995). Serum lipid and fat-soluble vitamin levels in primary sclerosing cholangitis. J. Clin. Gastroenterol..

[13] Sebode M., Peiseler M., Franke B., Schwinge D., Schoknecht T., Wortmann F., Quaas A., Petersen B.S., Ellinghaus E., Baron U. (2014). Reduced FOXP3(+) regulatory T cells in patients with primary sclerosing cholangitis are associated with IL2RA gene polymorphisms. J. Hepatol..

[14] Fisher S.A., Rahimzadeh M., Brierley C., Gration B., Doree C., Kimber C.E., Plaza Cajide A., Lamikanra A.A., Roberts D.J. (2019). The role of vitamin D in increasing circulating T regulatory cell numbers and modulating T regulatory cell phenotypes in patients with inflammatory disease or in healthy volunteers: A systematic review. PLoS ONE.

[15] Bowlus C.L., Arrive L., Bergquist A., Deneau M., Forman L., Ilyas S.I., Lunsford K.E., Martinez M., Sapisochin G., Shroff R. (2022). AASLD practice guidance on primary sclerosing cholangitis and cholangiocarcinoma. Hepatology.

[16] Trepo E., Ouziel R., Pradat P., Momozawa Y., Quertinmont E., Gervy C., Gustot T., Degre D., Vercruysse V., Deltenre P. (2013). Marked 25-hydroxyvitamin D deficiency is associated with poor prognosis in patients with alcoholic liver disease. J. Hepatol..

[17] Finkelmeier F., Kronenberger B., Zeuzem S., Piiper A., Waidmann O. (2015). Low 25-Hydroxyvitamin D Levels Are Associated with Infections and Mortality in Patients with Cirrhosis. PLoS ONE.

[18] Efe C., Kav T., Aydin C., Cengiz M., Imga N.N., Purnak T., Smyk D.S., Torgutalp M., Turhan T., Ozenirler S. (2014). Low serum vitamin D levels are associated with severe histological features and poor response to therapy in patients with autoimmune hepatitis. Dig. Dis. Sci..

[19] Paternostro R., Wagner D., Reiberger T., Mandorfer M., Schwarzer R., Ferlitsch M., Trauner M., Peck-Radosavljevic M., Ferlitsch A. (2017). Low 25-OH-vitamin D levels reflect hepatic dysfunction and are associated with mortality in patients with liver cirrhosis. Wien. Klin. Wochenschr..

[20] Finkelmeier F., Kronenberger B., Koberle V., Bojunga J., Zeuzem S., Trojan J., Piiper A., Waidmann O. (2014). Severe 25-hydroxyvitamin D deficiency identifies a poor prognosis in patients with hepatocellular carcinoma-a prospective cohort study. Aliment. Pharmacol. Ther..

[21] Bischoff-Ferrari H.A., Giovannucci E., Willett W.C., Dietrich T., Dawson-Hughes B. (2006). Estimation of optimal serum concentrations of 25-hydroxyvitamin D for multiple health outcomes. Am. J. Clin. Nutr..

[22] Kim W.R., Therneau T.M., Wiesner R.H., Poterucha J.J., Benson J.T., Malinchoc M., LaRusso N.F., Lindor K.D., Dickson E.R. (2000). A revised natural history model for primary sclerosing cholangitis. Mayo Clin. Proc..

[23] Bowlus C.L., Lim J.K., Lindor K.D. (2019). AGA Clinical Practice Update on Surveillance for Hepatobiliary Cancers in Patients With Primary Sclerosing Cholangitis: Expert Review. Clin. Gastroenterol. Hepatol..

[24] Stokes C.S., Volmer D.A., Grunhage F., Lammert F. (2013). Vitamin D in chronic liver disease. Liver Int..

[25] Iruzubieta P., Teran A., Crespo J., Fabrega E. (2014). Vitamin D deficiency in chronic liver disease. World J. Hepatol..

[26] Lim L.Y., Chalasani N. (2012). Vitamin d deficiency in patients with chronic liver disease and cirrhosis. Curr. Gastroenterol. Rep..

[27] Basile U., Napodano C., Pocino K., Barini A., Marino M., Santini S.A., Barini A., Stefanile A., Basile V., Calla C.A. (2019). Lack of association between Vitamin D status and free light chains profile with different chronic HCV-related liver and extrahepatic disorders. Eur. Rev. Med. Pharmacol. Sci..

[28] Goode E.C., Clark A.B., Mells G.F., Srivastava B., Spiess K., Gelson W.T.H., Trivedi P.J., Lynch K.D., Castren E., Vesterhus M.N. (2019). Factors Associated With Outcomes of Patients With Primary Sclerosing Cholangitis and Development and Validation of a Risk Scoring System. Hepatology.

[29] Yadlapati S., Judge T.A. (2021). Risk of Hepatobiliary-Gastrointestinal Malignancies and Appropriate Cancer Surveillance in Patients With Primary Sclerosing Cholangitis. Cureus.

[30] Jeon S.M., Shin E.A. (2018). Exploring vitamin D metabolism and function in cancer. Exp. Mol. Med..

[31] Markotic A., Kelava T., Markotic H., Silovski H., Mrzljak A. (2022). Vitamin D in liver cancer: Novel insights and future perspectives. Croat. Med. J..

[32] Casadei-Gardini A., Filippi R., Rimini M., Rapposelli I.G., Fornaro L., Silvestris N., Aldrighetti L., Aimar G., Rovesti G., Bartolini G. (2021). Effects of Metformin and Vitamin D on Clinical Outcome in Cholangiocarcinoma Patients. Oncology.

[33] Kornfeld D., Ekbom A., Ihre T. (1997). Survival and risk of cholangiocarcinoma in patients with primary sclerosing cholangitis. A population-based study. Scand. J. Gastroenterol..

[34] Ngu J.H., Gearry R.B., Wright A.J., Stedman C.A. (2011). Inflammatory bowel disease is associated with poor outcomes of patients with primary sclerosing cholangitis. Clin. Gastroenterol. Hepatol..

[35] Wiesner R.H., Grambsch P.M., Dickson E.R., Ludwig J., MacCarty R.L., Hunter E.B., Fleming T.R., Fisher L.D., Beaver S.J., LaRusso N.F. (1989). Primary sclerosing cholangitis: Natural history, prognostic factors and survival analysis. Hepatology.

[36] Siffledeen J.S., Siminoski K., Steinhart H., Greenberg G., Fedorak R.N. (2003). The frequency of vitamin D deficiency in adults with Crohn’s disease. Can. J. Gastroenterol..

[37] Fialho A., Fialho A., Kochhar G., Shen B. (2016). The presence of primary sclerosing cholangitis in patients with ileal pouch anal- anastomosis is associated with an additional risk for vitamin D deficiency. Gastroenterol. Rep..

[38] Grunhage F., Hochrath K., Krawczyk M., Hoblinger A., Obermayer-Pietsch B., Geisel J., Trauner M., Sauerbruch T., Lammert F. (2012). Common genetic variation in vitamin D metabolism is associated with liver stiffness. Hepatology.

